# Integrating single-cell omic techniques to resolve the spatio-temporal complexity of arbuscular mycorrhizal symbiosis

**DOI:** 10.1093/jxb/eraf404

**Published:** 2025-10-01

**Authors:** Gabriel Ferreras-Garrucho, Tania Chancellor, Uta Paszkowski

**Affiliations:** Crop Science Centre, Department of Plant Sciences, University of Cambridge, 93 Lawrence Weaver Rd, Cambridge CB3 0LE, UK; Crop Science Centre, Department of Plant Sciences, University of Cambridge, 93 Lawrence Weaver Rd, Cambridge CB3 0LE, UK; Crop Science Centre, Department of Plant Sciences, University of Cambridge, 93 Lawrence Weaver Rd, Cambridge CB3 0LE, UK; RIKEN, Yokohama, Kanagawa 230-0045, Japan; University of Cambridge, UK

**Keywords:** Arbuscular mycorrhizal symbiosis, cell-type specific tagging, live-cell imaging, plant-microbe interactions, single-cell omics, single-nucleus sequencing, spatial transcriptomics, spatio-temporal dynamics, translatomics

## Abstract

Arbuscular mycorrhizal symbiosis (AMS) is a ubiquitous and ancient interaction between plant root systems and fungi of the Glomeromycotina subphylum. The resulting relationship is mutually beneficial and deeply intimate, where the fungus intracellularly colonises root cortex cells to receive organic carbon and deliver minerals and water to the plant. Fungal colonisation of plant roots and cells is extremely dynamic and asynchronous across the root system. Development of symbiosis must, therefore, result from spatio-temporally fine-tuned molecular control mechanisms of both plant and fungus. Although the plant genetic program underpinning AMS has been extensively studied, little is known about its dynamic regulation across root cell layers and developmental stages of the association. Thus, many questions remain outstanding: how do different cell-types transcriptionally respond to AMS, how are distinct cell-type specific regulatory states coordinated, and what are the fungal transcriptional activities associated with discrete stages of root colonisation? The advent of single cell-based techniques now enables the high-resolution analysis to address these questions. In this review, we recapitulate the current knowledge on the spatio-temporal control of AMS, evaluate the relevance of existing spatial datasets to AMS research, and provide new perspectives for future study.

## Teaser

We explore recent methodological advancements in single cell technologies to unravel the spatio-temporal dynamics of arbuscular mycorrhizal symbiosis, focusing on cell-type-specific plant and fungal transcriptomes.

## Introduction

Roots serve as an extensive surface for the plant’s interaction with different soil-borne microorganisms. Continuous root growth and associated tissue differentiation fundamentally influence interactions with the rhizosphere inhabitants. Soil microorganisms, existing along a continuum from parasitism to mutualism, are highly diverse in their infection mechanisms (Zipfel and Oldroyd, 2017; Kawa and Brady, 2022). Plant pathogens infect roots using a range of cell-types, root zones, or root types as entry points (Tsai *et al.*, 2023). Commensal microorganisms, whether present in the soil, on the root surface, or inside the root (endophytic), are also influenced by the diversity of plant roots and their spatial patterning (Kawa and Brady, 2022). The same is true for symbiotic organisms, particularly for endosymbionts that reside inside the plant root (Genre *et al.*, 2020).

The most ubiquitous and ancient of such endosymbiotic interactions are mutualistic arbuscular mycorrhizal symbioses (AMS) between plant and fungi of the Glomeromycotina subphylum. The fungus contributes to the plant’s uptake of inorganic minerals, mainly phosphorus and nitrogen, in exchange for photosynthates, primarily sugars and lipids (Pfeffer *et al.*, 1999; Douds *et al.*, 2000; Jiang *et al.*, 2017; Keymer *et al.*, 2017; Luginbuehl *et al.*, 2017; Roth and Paszkowski, 2017; Wipf *et al.*, 2019). The evolutionary and ecological importance of this interaction cannot be overstated, having arisen in the first land plants around 450 million years ago, and contributing to improved plant nutrition in approximately 85% of the plant kingdom today (Pirozynski and Malloch, 1975; Kenrick and Strullu-Derrien, 2014; Strullu-Derrien *et al.*, 2018). The establishment of AM symbioses occurs rapidly, with the fungus extensively proliferating intra- and extraradically and repeatedly re-invading the root, leading to an asynchronous colonisation of the root. The key structures of the interaction are the transient intracellularly developing tree-like fungal feeding structures, the arbuscules, that facilitate the bi-directional exchange of nutrients between plant and fungus (Smith and Read, 2008).

Undoubtedly, such an extreme form of compatibility requires tight control from both partners, manifesting as characteristic patterns of gene expression and protein abundance (Pimprikar and Gutjahr, 2018). The anatomy and underlying genetic programs controlling AM symbioses have been extensively investigated. However, little is known about the spatio-temporal regulation of gene expression patterns required by the different plant cell types to enable engagement with the fungal symbiont across the distinct stages of development. Furthermore, even less is known about the genetic regulation and spatial patterning by the fungal partner due to the genetic intractability of arbuscular mycorrhizal fungi (AMF), their obligate biotroph lifestyle, and their aseptate hyphal networks.

In this review, we evaluate the current understanding of the regulation of AMS in space and time and explore the spatial complexity of roots as a site for biotic interactions. We highlight key outstanding questions that can now be addressed by newly developed, single-cell-based methodologies. To this end, we introduce and assess new high-resolution spatial omics techniques and propose a pipeline of methodologies which may be leveraged by the molecular plant-microbe community to advance our understanding of AMS and other root-microbe interactions at single-cell resolution.

## Spatio-temporal complexity of arbuscular mycorrhiza and roots as a surface for biotic interactions

**Fig. 1.**
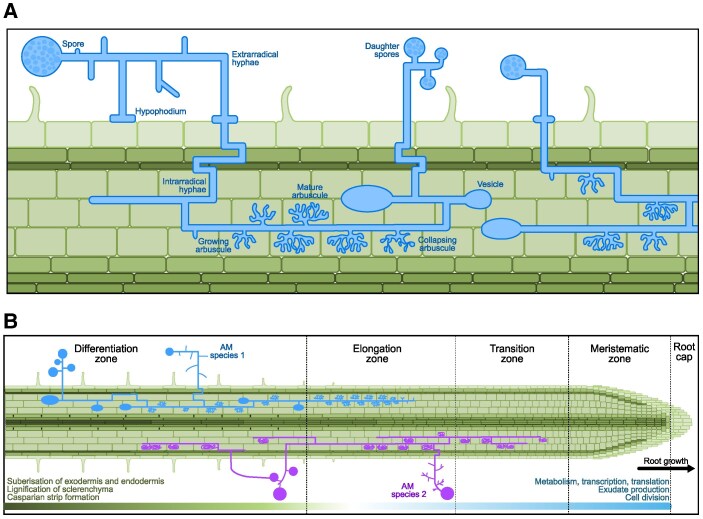
Spatio-temporal dynamics of arbuscular mycorrhizal symbiosis at diverging plant scales. (A) Zoomed-in general root colonised by arbuscular mycorrhizal fungi (AMF) is shown, showcasing the extra- and intraradical fungal structures and their spatial localisation within the root layers, as well as the asynchronous nature of the interaction, as distinct colonisation stages are present in the same root. (B) Detail of a general root with exodermis, sclerenchyma, and multiple cortical layers, highlighting the longitudinal organisation of the root zones from root cap to differentiation zone. Darker colouring of the exodermis, endodermis, sclerenchyma, and xylem indicates their increasing level of suberisation, lignification, and/or formation of Casparian strips going from the meristem to the differentiation zone. This is also displayed as a gradient on the bottom left of the box. Alongside and opposite this gradient, the increasing metabolic, transcriptional, and translational activity, as well as cell division and conceivably production and release of exudates, of the meristem versus the mature root is displayed on the bottom right of the box. Hypothetical patterns of colonisation by AMF penetrating through either the differentiation or elongation zones are displayed. Two different AMF species with differing intraradical colonisation structures (arbuscules and vesicles for species 1, hyphal coils for species 2) are shown.

Upon reaching the root surface, the fungal hyphae swell to form hypophodia from which they enter the root tissue. This may involve passage either in-between two epidermal cells (Demchenko *et al.*, 2004), or through the epidermal cell lumen, facilitated by the formation of a pre-penetration apparatus that guides the hyphae through the cell en route to the cortex (Genre *et al.*, 2005, 2008). The fungus extensively colonises the root cortex but does not invade endodermal cells. In the cortex, AM fungal hyphae spread longitudinally along the root length and initiate the formation of arbuscules, a hallmark of the association, through invagination of the plasma membrane of individual cortex cells. Arbuscules are formed by reiterative dichotomous hyphal branching inside plant host cells, extending from a trunk to high order branching at full arbuscule maturity (Smith and Read, 2008; Harrison, 2012), constantly surrounded by a plant derived peri-arbuscular membrane. Arbuscules are ephemeral structures which rapidly form and collapse over a period of hours to days, a process involving massive yet reversible changes of the host plant cell architecture (Harrison, 2012; Gutjahr and Parniske, 2013). Following sufficient nourishment by the plant host, the fungus produces lipid-storage vesicles, daughter spores, and extensive extraradical mycelia that initiate new infections on the same or neighbouring roots, resulting in the simultaneous presence of the different fungal structures (Smith and Read, 2008). This asynchronous development (Fig. 1A), considering both roots and hyphae are constantly growing, results in a remarkable level of spatio-temporal complexity. Furthermore, in the natural environment, different AMF species, as well as other mycorrhizal and endophytic fungi, can co-colonise the same root, harbouring differing structures that can potentially be functionally distinct (Smith *et al.*, 2000; Dickson, 2004; Jansa *et al.*, 2008).

AMF colonisation of the root system must be tightly linked with root anatomy and physiology (Fig. 1B). As roots continuously grow, the fungus readily colonises the young root tissue but never invades the meristematic region of the root. Root cells form a developmental gradient along the root’s longitudinal axis, reflecting the dynamic processes of cell division, elongation, differentiation, and specialization (Benfey and Scheres, 2000). Each zone offers a unique microenvironment, often comprising distinct signalling pathways and resources that shape microbial activity, significantly influencing the outcome of plant-microbial interactions (Kawa and Brady, 2022). For instance, root hairs, which are produced from the differentiation zone, have been shown to be an important microbial niche influencing the rhizosphere (Robertson-Albertyn *et al.*, 2017; Holz *et al.*, 2018; Quattrone *et al.*, 2024), and is well reported during the initial stages of rhizobial colonisation of legumes (Poole *et al.*, 2018). In addition, the root cap is known to be enriched with active microbes, acting as an initial path for colonisation of the epidermis and as a determinant of rhizosphere assembly (Liu *et al.*, 2021; Rüger *et al.*, 2023; Tsai *et al.*, 2023).

Radial barriers within the root also act as a key determinant of microbial colonisation (Kawa and Brady, 2022). Along the radial axis, root cell types are organised in concentric cylinders, with three main areas: a vascular bundle or stele, an endodermis, and several outer cell layers including cortex, exodermis, and epidermis, all of which vary according to species (Benfey and Scheres, 2000). Notably, the endodermis and exodermis act as essential barriers to nutrient/water transport, as well as microbial signal diffusion and invasion from other organisms, thanks to their suberisation and the endodermal lignified Casparian strip (Barberon, 2017; Doblas *et al.*, 2017; Barbosa *et al.*, 2019; Serra and Geldner, 2022). Consequently, unless infecting through the undifferentiated meristematic zone or at damaged sites including emerging lateral roots, root-infecting microbes must penetrate the many radial layers of the mature root, overcoming the exodermal and endodermal barriers of suberisation and lignification (Millet *et al.*, 2010; Maron, 2019; Zhou *et al.*, 2020; Tsai *et al.*, 2023). This explains why the elongation zone or lateral root emergence sites are preferred points of attack for pathogens, as the endodermal barrier is not yet established or is damaged/remodelled, respectively, in those areas (Czymmek *et al.*, 2007; Li *et al.*, 2017; Zhou *et al.*, 2020). However, a number of questions remain unresolved. For instance, can AMF infect the root preferentially through more ‘open’ parts of the root? Furthermore, can the presence of an exodermis and its degree of suberisation be somehow overcome during AMF colonisation?

AM fungi preferentially colonize lateral roots as opposed to the main tap root of dicotyledons or monocotyledonous crown roots (CR). This is possibly due to the younger and expanding tissue of lateral roots being less sturdy, aerenchymatic (Box 1), or lignified, and thus more amenable to fungal invasion (Hooker *et al.*, 1992; Gutjahr *et al.*, 2009). This has been particularly well reported for rice (*Oryza sativa*), where large lateral roots (LLRs) rapidly reach between 70% and 100% colonisation levels upon infection (Fiorilli *et al.*, 2015; Gutjahr *et al.*, 2015*b*). The rice-specific determinate fine lateral roots (FLRs), which lack a proper cortical cell layer that is separate from the exo/hypodermis, are perplexingly not susceptible to AMF (Gutjahr and Paszkowski, 2013; Watanabe *et al.*, 2020). Interestingly, in soybean (*Glycine max*), gene expression patterns suggest enhanced carbohydrate supply to lateral roots, which could explain why they are preferentially colonised by AMF (Blee and Anderson, 2002). Similarly, in *Medicago truncatula*, lateral roots are more transcriptionally responsive to fungal molecules (Kosuta *et al.*, 2003), and in *Petunia* the transporter of fungal attracting strigolactones PLEIOTROPIC DRUG RESISTANCE 1 (PDR1) is expressed in hypodermal passage cells of lateral roots only (Kretzschmar *et al.*, 2012). Overall, the existence of distinct root types or orders, each comprising different anatomies, physiologies, and responses to abiotic and biotic factors (Kawa and Brady, 2022), provides a further level of complexity to the interaction of AMF with plant roots.

Box 1. **Glossary**

- Aerenchyma is a specialized plant tissue with extensive intercellular air spaces, formed through programmed cell death or cell separation, facilitating gas exchange and oxygen transport in response to hypoxic or waterlogged conditions.
- Fluorescence-Activated Cell Sorting (FACS) is a specialized form of flow cytometry that utilizes fluorescent markers to identify and separate specific groups of cells based on their unique characteristics.
- Laser-Capture Microdissection (LCM) is a technique that uses a laser to precisely isolate and extract targeted microscopic tissue regions from their surrounding environment for further analysis.
- Dimensionality reduction is the process of simplifying high-dimensional data by reducing the number of features while preserving its essential patterns and structure. Common techniques include Principal Component Analysis (PCA) for linear transformations, and non-linear methods like Uniform Manifold Approximation and Projection (UMAP) and t-Distributed Stochastic Neighbor Embedding (t-SNE), which are often used for visualizing complex datasets in two or three dimensions.
- Supervised clustering is a method of analysis in single-cell omics which employs algorithms like random forests, support vector machines (SVM), or neural networks to group cells based on labeled features, while unsupervised clustering uses methods such as k-means, hierarchical clustering, or graph-based algorithms like Louvain or Leiden to identify patterns and cluster cells without prior labels.

## Genetic control of arbuscular mycorrhizal symbioses and its spatial patterning

The plant genetic program underpinning symbiosis development has been well studied (see Luginbuehl and Oldroyd, 2017; Choi *et al*., 2018; Chiu and Paszkowski, 2020; Paries and Gutjahr, 2023 for detailed reviews). Briefly, genetic control includes the biosynthesis of AMF attracting molecules such as strigolactones, recognition of fungal chitinaceous signals via receptor-like kinases or receptor-like proteins, nuclear calcium spiking, and activation of the common symbiosis signalling pathway (CSSP), named such as it is equally necessary for AM and for root nodulation symbiosis (RNS) in legumes (Kistner and Parniske, 2002; Parniske, 2004; Chiu *et al.*, 2018; Choi *et al.*, 2018). This pathway launches the activation of a suite of genes involved in AMS, from fungal accommodation to arbuscule formation and nutrient exchange, including well-studied nutrient transporters of phosphate, nitrogen, sugars, and lipids, as well as the symbiotic lipid biosynthetic programme (Luginbuehl and Oldroyd, 2017; Choi *et al.*, 2018). Environmental and developmental factors also have a strong influence on the plant regulation of AMS, including nutrient (Chiu and Paszkowski, 2019; Paries and Gutjahr, 2023) and phytohormonal signalling (McGuiness *et al.*, 2019; Hull *et al.*, 2021). Root permissibility to AMF colonisation is known to be induced under nutrient starvation conditions, associated with increased strigolactone release and transcriptional activation of symbiotic genes, and is hypothesised to be induced by signalling via the phosphate starvation pathway and karrikin receptor module (Gutjahr *et al.*, 2015*a*; Choi *et al.*, 2020; Shi *et al.*, 2021, 2022; Das *et al.*, 2022; Li *et al.*, 2022). Other phytohormonal signalling, particularly gibberellic acid signalling through DELLA proteins, has also been shown to influence AMS as well as RNS (Yu *et al.*, 2014; Pimprikar *et al.*, 2016; McGuiness *et al.*, 2019).

However, when and where the signalling components are required to drive genetic symbiosis programmes is largely unknown. The dynamic and asynchronous nature of AMS and arbuscule development has made it particularly challenging to address experimentally. Past research has relied on transcriptional and translational reporters, RNA in-situ hybridization, and laser-capture microdissection coupled with transcriptome analyses to obtain spatial information of the activity of the genetic components of AMS. In many cases, these are extrapolated from results in the legume-rhizobia symbiosis or wider plant-microbe interactions. Laser microdissection studies followed by either RT-qPCR or microarrays focused on capturing either early colonisation attempts or arbusculated cortical cells (Balestrini *et al.*, 2009; Gomez and Harrison, 2009, Preprint; Limpens, 2019). The studies were predominantly performed in *Medicago* (Siciliano *et al.*, 2007; Gomez *et al.*, 2009; Hogekamp *et al.*, 2011; Gaude *et al.*, 2012; Hogekamp and Küster, 2013), *Lotus japonicus* (Guether *et al.*, 2009; Giovannetti *et al.*, 2012, 2014), tomato (*Solanum lycopersicum*; Balestrini *et al.*, 2007; Fiorilli *et al.*, 2009), and rice (Campos-Soriano *et al.*, 2011; Roth *et al.*, 2018). Hogekamp and Küster (2013) conducted the most comprehensive study, performing microarray transcriptome analysis on epidermal and cortical cells captured underneath hypophodia, as well as epidermal, cortical, and arbusculated cells captured from mycorrhizal roots. Spatial patterning of gene expression was evident and distinct gene modules governing the sequential reprogramming of host roots could be detected, particularly genes activated specifically in arbusculated cells, including 42 AMS-activated transcription factors. However, this study did not dissect cells from non-colonised roots, which prevented proper differential gene expression analysis and distinguishing cell-type specific responses to AMF.

Plant transcriptional and translational reporters, particularly promoter-driven GUS (β-glucuronidase) staining, have been generated and characterised for a number of genes involved in arbuscular mycorrhizal symbiosis. These include nutrient transporters, signalling components in the CSSP, and genes involved in the regulation of AMS, such as phosphate starvation regulators. A selection of these is shown in Table 1. The result is an extensive, yet incomplete and perhaps biased, picture of AMS gene expression patterns. Most studies focus on gene induction in colonised tissue and the arbusculated cell, whereas more nuanced expression patterns at early colonisation stages or in non-colonised roots are often overlooked. Therefore, little is known about the spatial patterning of many AMS-associated genes, particularly in non-colonised tissues, in different root zones and root types, or at distinct stages of plant development. Pioneering work in the spatial patterning of gene expression throughout root development (Birnbaum *et al.*, 2003; Brady *et al.*, 2007) was mostly performed in the non-host for AMF *Arabidopsis thaliana*, and therefore the datasets could not be investigated for AMS-induced genes or regulators. Many questions remain open regarding the spatial patterning of gene expression during AMS in distinct cell-types and zones, and its functional relevance (Box 2).

Box 2. **Outstanding questions regarding the spatial dynamics of arbuscular mycorrhizal symbioses**

- How does the fungus regulate gene expression during AMS development? How is fungal mRNA and protein deployment coordinated in the continuum of the mycelium? Which hyphal areas are more transcriptionally active?
- How do different plant root types, zones, and cell-types respond to AMS? Are there transcriptional, epigenetic, or other factors that prime roots, root types, or zones to be colonised by AMF? How is intraradical accommodation of diverse AMS structures inside the root coordinated dynamically as the root continuously grows and differentiates? Is it possible to establish developmental trajectories for these stages of plant-fungal interaction through single-cell or spatial omics as they occur asynchronously?
- Do all arbusculated cells share the same expression profile throughout their lifecycle, or does it vary according to the root type, zone, or even individual cell? How do cells prepare to contain arbuscules, do bulk methods overlook genes required to prime cells for arbusculation or to be in close contact to fungal hyphae? What changes occur in non-colonised tissues like the endodermis and stele?
- Where are AM signalling components expressed, where do they initiate signalling, and is this signalling cell-autonomous or not? Are arbuscule-specific genes such nutrient transporters truly exclusive or do they have some basal expression in tissue not directly colonised? Do AM induced components such as phosphate starvation regulators maintain their basal expression patterns upon colonisation, or is there a complete repatterning of gene expression? How do these changes return to homeostasis after the collapse of the arbuscule?

**Table 1.** Selection of genes induced, involved in regulation, or with known activity in arbuscular mycorrhizal symbiosis that have a resolved spatial expression pattern through transcriptional/translational reporters or in-situ hybridization

Gene	Species	Root spatial pattern	Conditions tested	Technique	Reference
*Arbuscule specific*					
*LYK1, ARK1*	*Oryza sativa*	Arbusculated cells	AMF inoculation	GUS transcriptional reporter	(Roth *et al.*, 2018)
*PT11, PT13*	*Oryza sativa*	Arbusculated cells	AMF inoculation	GUS transcriptional reporter	(Yang *et al.*, 2012)
*PT11, AM1, AM3*	*Oryza sativa*	Arbusculated cells	AMF inoculation	In-situ hybridization	(Gutjahr *et al.*, 2008)
*PT11*	*Oryza sativa*	Arbusculated cells	AMF inoculation	FP translational fusion	(Kobae and Hata, 2010)
*PT4*	*Medicago truncatula*	PAM of arbusculated cells	AMF inoculation	GUS transcriptional reporter, FP translational fusion	(Harrison *et al.*, 2002; Pumplin and Harrison, 2009)
*ADK1*	*Oryza sativa*	Arbusculated cells	AMF inoculation	*Promoter:GUS*	(Guo *et al.*, 2022)
*SWEET6*	*Glycine max*	PAM of arbusculated cells	AMF inoculation	FP translational fusion	(Zheng *et al.*, 2024)
*NPF4.5*	*Oryza sativa*	Arbusculated cells	AMF inoculation	*Promoter:GUS*	(Wang *et al.*, 2020)
*AMT3;1*	*Zea mays*	PAM of arbusculated cells	AMF inoculation	FP translational fusion	(Hui *et al.*, 2022)
*HA1*	*Medicago truncatula*	PAM of arbusculated cells	AMF inoculation	FP translational fusion	(Krajinski *et al.*, 2014)
*HA8*	*Solanum lycopersicum*	PAM of arbusculated cells	AMF inoculation	FP translational fusion	(Liu *et al.*, 2016)
*Enriched in colonised tissue and/or arbusculated cells*					
*SWEET1b*	*Medicago truncatula*	Colonised cortex, stronger in arbusculated cells, root tip	AMF inoculation	*Promoter:GUS*	(An *et al.*, 2019)
*SWEET2c, 7a and 12a*	*Solanum tuberosum*	*SWEET2c and 12* in root tip and cortex, *SWEET7a* expressed ubiquitously, induced under AMS mainly in arbusculated cells	AMF inoculation	*Promoter:GUS*	(Manck-Götzenberger and Requena, 2016)
*STR, STR2*	*Medicago truncatula*	Low expression in stele, stronger in arbusculated cells	AMF inoculation	*Promoter:GUS*	(Zhang *et al.*, 2010)
*RAM1, RAM2*	*Medicago truncatula*	*RAM1* in colonised roots, *RAM2* in arbusculated cells	AMF inoculation	*Promoter:GUS*	(Gobbato *et al.*, 2013)
*ZAS, ZAS2*	*Oryza sativa*	Ubiquitous, stronger in exodermis, under P starvation and in arbusculated cells	AMF inoculation, Pi starvation	*Promoter:GUS, in situ* hybridization	(Votta *et al.*, 2022; Ablazov *et al.*, 2023)
*RAM1*	*Lotus japonicus*	Colonised roots, arbusculated cells	AMF inoculation	*Promoter:GUS*	(Pimprikar *et al.*, 2016)
*RAD1*	*Medicago truncatula*	Cortex cells proximal to *P. palmivora* hyphae	*P. palmivora* inoculation	*Promoter:GUS*	(Rey *et al.*, 2017)
*RAD1*	*Lotus japonicus*	Expressed in low P, mostly in vasculature and in AMF-colonised tissue	AMF inoculation, Pi starvation	*Promoter:GUS*	(Xue *et al.*, 2015)
*RAM1*	*Lotus japonicus*	Lateral root primordia, meristem, colonised cortex and arbusculated cells	AMF inoculation, Pi starvation	*Promoter:GUS*	(Xue *et al.*, 2015)
*PHR2*	*Oryza sativa*	Ubiquitous, induced by low Pi, higher in arbusculated cells	AMF inoculation, Pi starvation	*Promoter:GUS*	(Shi *et al.*, 2021)
*SPX1, 3*	*Medicago truncatula*	Cortex and epidermis, repressed by high Pi, strong in arbusculated cells	High vs low Pi	*Promoter:GUS, Promoter:NLS-FP*	(Wang *et al.*, 2021*b*)
*NSP1, NSP2, D27, MAX1*	*Medicago truncatula*	*NSP1* and 2 in root tip and emerging laterals, D27 and *MAX1* in vasculature, root tip and emerging laterals, *NSP1* in early colonisation, *NSP2*, *D27* and *MAX1* in colonised tissue.	AMF inoculation	*Promoter:GUS,* GUS translational fusion	(Guillotin, 2016)
*Not enriched in colonised tissue or not tested for AMS*					
*CASTOR, POLLUX*	*Lotus japonicus*	Ubiquitous, strongest at growing tips.	Rhizobia inoculation	*Promoter:GUS*	(Charpentier *et al.*, 2008)
*NFR5*	*Glycine max*	Ubiquitous, strong in stele, decrease with age, increased in young nodules	Rhizobia inoculation	*Promoter:GUS*	(Indrasumunar *et al.*, 2010)
*NFR5, CERK1*	*Oryza sativa*	Epidermis and cortex	Seedlings	*Promoter:GUS, in situ* hybridization	(He *et al.*, 2019)
*NSP2*	*Lotus japonicus*	Ubiquitous, stronger upon rhizobia infection in epidermis and developing nodule	Rhizobia inoculation	*Promoter:GUS*	(Kang *et al.*, 2014)
*SMAX1, SMXL6,7,8; MAX2, D14, KUF1*	*Arabidopsis thaliana*	Vasculature of mature roots, root cap, emerging lateral primordia	Normal growth	*Promoter:GUS*	(Shen *et al.*, 2007; Chevalier *et al.*, 2014; Soundappan *et al.*, 2015; Sepulveda *et al.*, 2022)

PAM, peri-arbuscular membrane; AMF, arbuscular mycorrhizal fungi; FP, fluorescent protein; Pi, inorganic phosphorus.

Spatial functional assays are powerful but even less common, with the only example in the RNS field being that of Hayashi *et al*., 2014. Here, a set of nodulation regulators were expressed in *Lotus japonicus* using epidermal, cortical, or ubiquitous promoters. Upstream common symbiosis genes, including receptor kinases and genes required for nuclear calcium spiking, in the epidermis were found to be sufficient for successful nodule formation. Conversely, downstream transcriptional activators were required in both epidermis and cortex for proper nodulation, although potentially under different regulation as they require differential upstream components. Studies on particular mutations in *SYMRK (SYMbiosis Receptor-like Kinase)* showed that the epidermal and cortical functions of this receptor in nodulation could be separated (Kosuta *et al.*, 2011; Saha *et al.*, 2016). Importantly, both these studies highlight how components of the same signalling pathway can be required in different tissues, or how even the same protein can have distinct functions in different cell-types. Though high-resolution transcriptional studies are crucial for the investigation of AMS spatial patterning, additional functional studies, such as those suggested here, are required to distinguish secondary association from primary functional requirement.

Particularly little is known about the spatial regulation of fungal gene expression. Genetic studies are challenging as successful stable transformation of AMF has not been reported, and as AMF species are obligate biotrophs that can only be cultured in the presence of roots (Young, 2015). Consequently, most of the cumulated knowledge on the fungal side is on identification of nutrient transporters (Duan *et al*., 2024 and citations therein), as well as effector proteins (Kloppholz *et al.*, 2011; Sȩdzielewska Toro and Brachmann, 2016; Wang *et al.*, 2021*a*; Aparicio Chacón *et al.*, 2023, 2024; Teulet *et al.*, 2023; Betz *et al.*, 2024). Due to the genetic intractability of the fungus, the only spatial studies have relied on RNA in-situ hybridization or heterologous expression (with the latter only applicable for studying subcellular localisation), showing, for instance, the expression of effectors, phosphate transporters, or copper exporters in the arbuscule, intraradical hyphae, and/or vesicles (Wang *et al.*, 2021*a*; Xie *et al.*, 2022; Teulet *et al.*, 2023; Gómez-Gallego *et al.*, 2024). Glomeromycotina fungi are particularly ‘anatomically’ interesting, being aseptate and therefore made of a continuous cytoplasm that encompasses all structures from spores to arbuscules and vesicles, from extraradical to intraradical and intracellular colonising fungal structures. The genetic regulation of this system is mostly unknown and must highly depend on nuclear dynamics and movement to resolve the transcriptional state of different hyphal areas (Schüßler *et al.*, 2001; Xiang, 2018).

## Leveraging single-cell/nuclei and spatial transcriptomics for AMS research

**Fig. 2.**
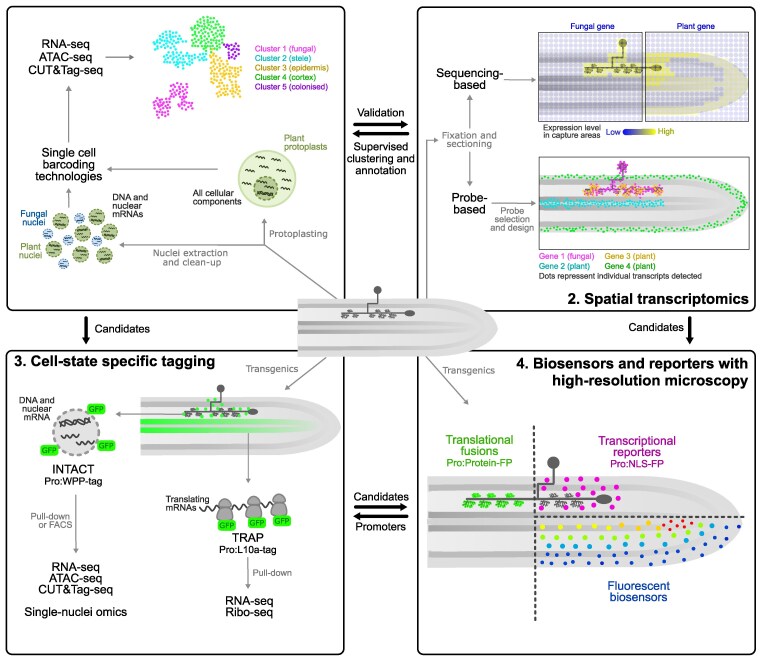
A pipeline for combining high-resolution spatial technologies to resolve the complexity of arbuscular mycorrhizal symbiosis (AMS). Each panel from 1 to 4 represents a stage in the proposed pipeline using a group of similar spatial technologies. Panel 1 represents single-cell and single-nuclei omics: tissue of interest can be subjected to either protoplasting or nuclei extraction (followed by either filtering or fluorescence-activate cell sorting, FACS, for clean-up) The protoplasts or nuclei are then individually barcoded using commercial or home-made technologies resulting in RNA-seq, ATAC-seq (Assay for Transposase-Accessible Chromatin), or other type of libraries such as CUT&Tag-seq (Cleavage Under Targets and Tagmentation) that are sequenced using common methods. Resulting data is processed through increasingly streamlined bioinformatic approaches which result in dimensionality reduction, clustering (represented in the figure), cell-type annotation, and further downstream analyses such as differential gene expression (DGE). Panel 2 represents spatial transcriptomics: tissue of interest is fixed, sectioned, and prepared according to the technology to be used and manufacturer’s instructions, which can be either sequencing-based or probe-based/multiplexed-FISH (fluorescent *in-situ* hybridization). Sequencing-based techniques result in data similar to single-cell/nuclei with transcript counts for capture areas (instead of individual cells), which can then be subjected to similar analyses. Probe-based methods generate high-resolution imaging data where individual transcripts are detected as fluorescent signal. These images can then be subjected to cell segmentation to create single-cell-type gene count datasets. Panel 3 represents cell-state specific tagging techniques: cell-type specific or inducible promoters can be used to express tags in a particular tissue, cell-type, or cell-state of interest, either free in cytoplasm, nucleus, or other organelles, for example by fusing to a nuclear-envelope protein such as WPP (INTACT, Isolation of Nuclei TAgged in specific Cell Types) or ribosomal protein such as L10a (TRAP, Translating Ribosome Affinity Purification). Once transgenic material is generated, tagged cells or organelles can be pulled-down using anti-tag antibodies to then create libraries; RNA or Ribo-seq for TRAP, and RNA, ATAC, or CUT&Tag-seq for INTACT. INTACT lines with a fluorescent tag can be used for nuclei-extraction followed by FACS to select for positive nuclei and then create single-nuclei libraries. Panel 4 represents the use of fluorescent biosensors, transcriptional (for instance, expression of a nuclear-tagged fluorescent protein, NLS-FP, under a promoter of interest) and translational reporters (fusion of a protein of interest to a fluorescent protein, FP) for high-resolution microscopy, as well as other functional analyses, which require generation of transgenic material.

Single-cell and single-nuclei sequencing, in combination with spatial transcriptomics technologies, enable the study of gene expression, epigenomics, and cellular heterogeneity at an unprecedented resolution (Vandereyken *et al.*, 2023; Islam *et al.*, 2024). In single-cell/nuclei sequencing, individual cells or nuclei are isolated using microfluidics or droplet-based platforms, allowing the study of gene expression at the individual cell or nucleus level, thereby providing insights into cellular heterogeneity within tissues. Spatial transcriptomics, on the other hand, combines gene expression analysis with spatial location information within tissue samples. Here, the tissue is sectioned into thin slices, and sections are either hybridized with target-specific fluorescent probes (probe-based method) or captured at spatially barcoded sites (sequencing-based method) (Fig. 2). Single-cell/nuclei methods have been applied to a number of plant species, tissues, and experimental conditions, with the number of studies growing exponentially in the last few years (Grones *et al.*, 2024; Sun *et al.*, 2024 and citations therein). The first few datasets for plant spatial transcriptomics are now starting to emerge, in many cases in combination with single-cell/nuclei sequencing (Xia *et al.*, 2022; Guillotin *et al.*, 2023; Bawa *et al.*, 2024; Serrano *et al.*, 2024*a*; Nobori *et al.*, 2025). Their application to spatially profile mycorrhizal roots, including transcripts from both plant and fugus as pioneered by Serrano *et al.*, 2024*a*, holds great promise for addressing many of the unanswered questions in the field (Box 2). For instance, spatial transcriptomics could help investigate how fungal transcripts are spatially patterned in the continuum of the intraradical mycelium, and single-cell sequencing could elucidate the developmental trajectories of distinct cell-types during colonisation as well as the arbuscule life-cycle, from formation to collapse.

### Single-cell and single-nuclei omics resolve cellular dynamics at high resolution

Single-cell/nuclei transcriptomics (scRNA-seq) is quickly becoming a major technology in plant sciences, as evidenced by the growing literature on methodologies, analysis pipelines, and databases (McFaline-Figueroa *et al.*, 2020; Cervantes-Pérez *et al.*, 2022; Cao *et al.*, 2023; Chen *et al.*, 2023; Grones *et al.*, 2024; He *et al.*, 2024; Jia *et al.*, 2024). The available toolkit spans four key approaches: manual/FACS isolation-based (SMART-seq2, CEL-seq2, MARS-seq), droplet-based (10x Genomics Chromium, Drop-seq, PIP-seq), micro/nanowell-based (iCELL8, BD Rhapsody), and combinatorial indexing (SCI-seq, ParseBio Evercode) (Ding *et al.*, 2020; McFaline-Figueroa *et al.*, 2020; Chen *et al.*, 2023; Grones *et al.*, 2024; De Simone *et al.*, 2025; Nobori, 2025). Notably, manual/FACS-based methods achieve single-cell or -nucleus resolution through physical isolation into individual wells instead of barcoding-based multiplexing as the rest. Among these, 10x Genomics Chromium is most widely used in plant research due to its high throughput, ease of use, and compatibility with diverse tissues and species (Grones *et al.*, 2024; Sun *et al.*, 2024). Other platforms such as BD Rhapsody (Zong *et al.*, 2022; Tenorio Berrío *et al.*, 2025), iCELL8 (Sunaga-Franze *et al.*, 2021) and SMART-seq2 (Wang *et al.*, 2022) have also been successfully applied in plants. Combinatorial indexing, offering scalability and low cost, shows great promise for future applications (Tu *et al.*, 2022; Swift *et al.*, 2024; Zhang *et al.*, 2024).

The choice between nuclei or whole cells profiling remains debated. Single-cell methods generally capture more transcripts, including cytoplasmic and organellar, but require protoplasting which induces stress and can bias cell-type representation. Single-nucleus methods avoid these issues, are compatible with frozen tissues, and enrich for nascent transcripts but exclude organellar and cytoplasmic RNAs (McFaline-Figueroa *et al.*, 2020; Farmer *et al.*, 2021; Cervantes-Pérez *et al.*, 2022; Guillotin *et al.*, 2023; Adema *et al.*, 2024; Islam *et al.*, 2024). Overall, both approaches are valid and yield highly correlated data (Guillotin *et al.*, 2023). The choice depends on the biological system and research question. For plant–fungal interactions, single-nucleus profiling is preferred due to its compatibility with capturing both plant and fungal nuclei and the impracticality of co-protoplasting plant cells and fungal syncytia (Serrano *et al.*, 2024*a*, 2024*b*; Somoza *et al.*, 2024).

The technological toolkit of single-cell is also rapidly expanding with the advent of single cell multi-omics, including single-cell genomics (DNA-seq), epigenomics (Assay for Transposase-Accessible Chromatin, ATAC-seq, Bisulfite Sequencing, BS-seq; Chromatin ImmunoPurification, ChIP-seq; Chromosome Conformation Capture, Hi-C-seq; Cleavage Under Targets and Tagmentation, CUT&TAG-seq), proteomics, and metabolomics (Clark *et al.*, 2021; Baysoy *et al.*, 2023; Vandereyken *et al.*, 2023). These techniques can be applied individually or in combination, resulting in, for instance, paired transcriptomic and epigenomic sequencing data for each cell sampled (Baysoy *et al.*, 2023; Vandereyken *et al.*, 2023). The integration of such techniques is being applied in plant sciences, particularly the combination of scRNA-seq with scATAC-seq to uncover chromatin accessibility and associated transcriptional output, with results in *Arabidopsis*, rice, sorghum (*Sorghum bicolor*), maize (*Zea mays*), *Catharanthus roseus* and cotton (*Gossypium hirsutum*) (Dorrity *et al.*, 2021; Farmer *et al.*, 2021; Marand *et al.*, 2021*a*, 2021*b*; Li *et al.*, 2023; Mendieta *et al.*, 2023, 2024; Wang *et al.*, 2023; Swift *et al.*, 2024; Nobori *et al.*, 2025).

Recently produced plant single-cell atlases enable gene expression queries at cellular resolution in diverse tissues and developmental stages (Lee *et al.*, 2025; Zhang *et al.*, 2025). Extending these atlases to additional mycorrhizal-relevant species would enable exploration of targets involved in AMS, such as receptor-like kinases required for fungal perception, CSSP components, and regulators of AMS permissibility, providing insight into their spatial distribution within colonized roots, across plant development, and in distal tissues.

Single-cell/nuclei studies focusing on plant-microbe interactions confirm that responses to biotic factors are highly cell-type specific. In Arabidopsis, immune receptor families accumulate differentially in the vasculature in response to fungal infection (Tang *et al.*, 2023), and systemic acquired resistance genes are induced in phloem companion cells in response to *Pseudomonas syringae* (Nobori *et al.*, 2025). In legumes, scRNA-seq has revealed the transcriptomic and metabolic cellular heterogeneity in nodules (Ye *et al.*, 2022; Liu *et al.*, 2023*b*; Cervantes-Pérez *et al.*, 2024; Pereira *et al.*, 2024), as well as cell-type specific responses to Nod factors (Liu *et al.*, 2023*b*). Similarly for AMS, Serrano *et al.,* 2024a carried out the first single-nuclei sequencing of a mycorrhizal association between *Medicago truncatula* and *Rhizophagus irregularis*. This pioneering study confirmed the applicability of single-nuclei sequencing for the study of AMS, resulting in the validation of spatial expression patterns of known plant AMS markers (e.g. *Phosphate Transporter 4*, *FatM*, *Reduced Arbuscular Mycorrhization 2*), while also providing new candidates for further research. Unfortunately, fungal transcripts were not recovered via 10x Genomics Chromium (Serrano *et al.*, 2024*a*).

Recovering transcripts from both host and microbe remains a major challenge in single-cell plant–microbe studies. Most current technologies rely on polyA capture, limiting bacterial transcript detection (Zhu *et al.*, 2023; Serrano *et al.*, 2024*b*). Tools like micro-SPLiT, ProBac-seq and PETRI-seq address this but remain untested in plants (Blattman *et al.*, 2020; Kuchina *et al.*, 2021; McNulty *et al.*, 2023). In principle, dual plant–fungal single-nucleus profiling is feasible with polyA-based methods, but differences in nuclear size and composition, and distinct library prep and sequencing considerations, require significant protocol optimization. These technical hurdles likely contribute to the scarcity of mycorrhizal single-cell studies. Notably, root single-cell approaches were pioneered in non-mycorrhizal Arabidopsis root tips (Denyer *et al.*, 2019; Zhang *et al.*, 2019; Shahan *et al.*, 2022). In contrast, mature roots of AM-host species grown in substrate, as required to sustain AM colonisation, present additional challenges such as thickened root layers (e.g. suberized exodermis in tomato or lignified sclerenchyma in rice), which impede protoplast and nuclei isolation (Kawa and Brady, 2022; Adema *et al.*, 2024). Furthermore, the intimate and intricate plant-fungal connection, particularly in the arbusculated cell, might further hinder cell separation and nuclear extraction. Despite these hurdles, expanding single-cell datasets of mycorrhizal roots would be invaluable, for instance to dissect transcriptional trajectories during arbuscule development and degeneration, and thus providing long-sought-after marker genes for these distinct stages. Future work should also expand the range of plant–fungal interactions studied to uncover shared and distinct features of host recognition across pathogens, commensals, and symbionts. Additionally, the separate study of distinct root zones and root types could enable the integration of root development alongside asynchronous fungal growth.

### Spatial transcriptomics brings the spatiotemporal context back

While single-cell approaches excel at high-throughput profiling, cell dissociation discards the spatiotemporal context of the cell, and the data produced are sparse and have high levels of dimensionality. This requires dimensionality reduction and unsupervised clustering, followed by cell-type annotation based on known marker gene expression. However, unsupervised clustering emphasizes dominant variation, potentially overlooking subtle but biologically relevant differences, and annotation is often hindered in non-model species lacking well-characterized marker genes, necessitating laborious experimental validation (McFaline-Figueroa *et al*., 2020; Adema *et al*., 2024; Islam *et al*., 2024).

Both these issues can be elegantly solved using spatial transcriptomics which preserves the spatial context of gene expression and permits supervised dimensionality reduction and clustering, resulting in more refined and biologically relevant clusters (Adema *et al.*, 2024). Spatial transcriptomic technologies can be divided into two kinds: untargeted or sequencing-based (10x Genomics Visium, HD-Spatial Transcriptomics, Stereo-Seq) relies on spatially indexed cDNA barcoding that allows for downstream recovery of the spatial localisation of each transcript. Targeted or probe-based (10x Genomics Xenium, MERFISH, Molecular Cartography, PHYTOMap) uses multiplexed hybridization technologies to visualize hundreds to thousands of transcripts in the tissue (Xia *et al.*, 2022; Nobori *et al.*, 2023; Bawa *et al.*, 2024; Serrano *et al.*, 2024*b*). These techniques are also being expanded into spatial multi-omics, including genomic, epigenomic, and proteomic data (Vandereyken *et al.*, 2023).

The main limitation of spatial transcriptomics technologies is its resolution. For example, 10x Genomics Visium has a spatial resolution of 55 μm, hence not of single-cell resolution in plant tissues (Serrano *et al.*, 2024*a*). In addition, most spatial techniques require very thin sections, except for the whole-mount PHYTOMAP (Nobori *et al.*, 2023), so only relatively abundant genes are detected, with many low abundance transcripts being missed (Chen *et al.*, 2023; Serrano *et al.*, 2024*b*). Furthermore, these techniques require intensive sample pre-processing and are normally very costly (Adema *et al.*, 2024). Thus, single-cell/nuclei approaches remain unmatched for high-throughput profiling of thousands to millions of cells, while spatial transcriptomics enables supervised clustering, improved annotation and validation of single-cell datasets, and addresses specific biological questions, such as in plant–AMF interactions (Adema *et al.*, 2024; Serrano *et al.*, 2024*b*; Somoza *et al*., 2024).

Spatial transcriptomics is particularly valuable for studying plant–microbe interactions as it enables capture of both plant and microbial transcripts and bypasses the challenges of nuclei/protoplast isolation. This is especially relevant for AM research, where the genetic intractability of AMF and limited knowledge of spatial patterning of gene expression would severely limit annotation of single-nuclei clusters. Spatial approaches have already been applied in several plant-microbe models: MERFISH in Arabidopsis leaves under pathogen attack enabled spatial imputation of transcriptomes and chromatin landscapes (Nobori *et al.*, 2025); spatial meta-transcriptomics has captured bacterial and eukaryotic transcripts simultaneously in Arabidopsis (Saarenpää *et al.*, 2024); and Stereo-seq guided single-nuclei clustering in soybean nodules (Liu *et al.*, 2023*a*). Serrano *et al.*, 2024a used Visium alongside snRNA-seq to profile *Medicago* AMF colonised roots, which allowed capture of both plant and fungal transcripts, which was not possible using single-nuclei sequencing. This provided spatial resolution to known *R. irregularis* transcripts induced during colonisation, such as nutrient transporters and effectors, as well as new candidates, therefore providing an immensely useful resource for further investigation. However, the 55 μm resolution of Visium did not allow for mapping of transcripts to individual fungal structures and thus could not carefully examine plant and fungal gene expression through fungal development. Explorative spatial transcriptomics at single-cell resolution would be an extremely desirable advancement for AM research, but will depend on the development of sequencing methodologies with both improved transcript capture and higher spatial resolution.

## Integrating further high-resolution spatial techniques to resolve single-omic data

Single-cell and spatial transcriptomics approaches are limited by low sequencing depth, therefore potentially overlooking low abundance transcripts such as transcription factors or signalling components (Adema *et al.*, 2024). Moreover, as previously mentioned, some tissues, like mycorrhizal roots, may pose added challenges for processing (protoplasting, nuclei extraction, or sectioning), transcript capture, and sequencing. Also, rare cell types require deeper—and more costly—sequencing, which might be the case for some fungal structures or plant cells in close interaction with AMF (e.g. the arbusculated cell) (Adema *et al.*, 2024). Lastly, while these methods reveal transcript localization and abundance, functional validation requires complementary approaches. We outline several complementary research methodologies below (Fig. 2).

### Cell-state specific tagging of nuclei, ribosomes, and other cellular components to resolve gene dynamics with spatio-temporal resolution

Cell-state specific tagging appears as an elegant and simple technology to overcome the issues on cost and scarcity of data (Adema *et al.*, 2024). This category includes methods by which a particular tissue, cell-type, or cell-state of interest is tagged, normally through expression of a protein tag such as a fluorescent protein under a specific promoter. This tag can be expressed in the whole cell or in a particular subcellular compartment by fusion with a localisation signal or protein. The tag then allows for pull-down or selection of the cell or organelle of interest via fluorescence-activated cell sorting (FACS), magnetic beads, or other similar methodologies (Fröschel and Dröge-Laser, 2023). Examples of this include INTACT (Isolation of Nuclei TAgged in specific Cell Types), where nuclei of specific cell-types are tagged through fusion of a nuclear-envelope-targeting domain to GFP and Biotin Ligase Recognition Peptide (Deal and Henikoff, 2011; Reynoso *et al.*, 2018), and TRAP (Translating Ribosome Affinity Purification), where ribosomes are tagged by fusion of a ribosomal protein with GFP (Heiman *et al.*, 2014; Fröschel and Dröge-Laser, 2023).

A crucial advantage of these techniques is the use of diverse promoters, such as cell-type specific or inducible, which not only give spatial information, but can also provide temporal and developmental resolution, or be induced in response to a specific abiotic or biotic stimulus (Traubenik *et al.*, 2021; Fröschel and Dröge-Laser, 2023). It is therefore possible to extract transcripts exclusively from cells that are interacting with a biotic partner, such as AMF (You *et al.*, 2023). Another advantage is that some of these techniques can be combined with single-cell/nuclei omics to enrich for the cell-type or cell-state of interest while maintaining single-cell resolution. This combined approach was applied in Arabidopsis to profile lowly abundant phloem and companion cells (Otero *et al.*, 2022). This could be employed to profile similarly scarce or difficult to extract cell-types in AMS, such as the arbusculated cell.

The main issues associated with these techniques are the requirement of marker genes, the selection and design of promoters from these markers, and the costly and laborious generation of transgenic lines. The first is becoming less concerning as single-cell and spatial omic datasets are increasingly available (Yan *et al.*, 2022; Mendieta *et al.*, 2023; Islam *et al.*, 2024; Jia *et al.*, 2024; Chau *et al.*, 2025). The design of promoters from candidates can be guided through knowledge of epigenomic data, *cis*-regulatory sites, and transcription factor binding regions (Jores *et al.*, 2021; Schmitz *et al.*, 2022; Brooks *et al.*, 2023). An elegant solution would be the introduction of knock-ins of the desired tags in the native gene, although this could affect plant physiology and is only feasible in some cases (Rozov *et al.*, 2022; Schreiber *et al.*, 2024). Another future avenue is the design of synthetic promoters and gene circuits to drive gene expression in a more controlled fashion, although these technologies are still in development, particularly for non-model species (Brophy *et al.*, 2022; Jores *et al.*, 2021; Zhong *et al.*, 2023). Data from previous research on AMS in a wide range of species could be mined for candidate promoters for this purpose, such as those of arbuscule-specific nutrient transporters (Table 1).

INTACT and TRAP can be combined with transcriptomic, genomic, epigenomic, and even proteomic analyses. In the latter case, the use of proximity labelling paired with mass spectrometry can be specified to cell-types, states, or organelles through choice of specific promoters or localisation tags (Mair *et al.*, 2019; Xu *et al.*, 2025). Through INTACT, it is possible to investigate the nuclear transcriptome, genome, and epigenome through DNA, ChIP, CUT&TAG, BS or ATAC-seq (Deal and Henikoff, 2011; Reynoso *et al.*, 2018; Chongtham *et al.*, 2021). TRAP captures ribosome-associated transcripts, allowing the study of the translatome and providing a snapshot of active translation, as well as enabling ribosome footprinting (Ribo-seq) to investigate RNA dynamics at codon resolution (Traubenik *et al.*, 2021; Fröschel and Dröge-Laser, 2023). Together, INTACT and TRAP offer a powerful toolkit for investigating plant molecular regulation from chromatin state to translational control, all with cell-type specificity. Translational reprogramming has been found as an essential step in immune regulation (Xu *et al.*, 2017; Traubenik *et al.*, 2021; Wu *et al.*, 2024), and similar regulatory mechanisms may be involved during mycorrhizal colonisation, warranting further investigation.

INTACT and TRAP have already been applied to plant-microbe interactions. Fröschel *et al*., 2021 used TRAP-seq with cell-type-specific promoters targeting tissues colonised by commensal, pathogenic, and beneficial microbes, showing that translational responses reflect distinct colonisation strategies. You *et al*., 2023 used effector-inducible INTACT to profile transcriptomic changes in pathogen-infected cells, uncovering a novel virulence mechanism involving host osmosignalling. In soybean, cortex-specific TRAP-seq revealed early transcriptional responses to rhizobia and identified new candidate genes (Song *et al.*, 2022). In *Medicago*, a constitutive TRAP line highlighted widespread uncoupling between transcription and translation, and uncovered hundreds of regulated protein-coding and long noncoding RNAs (Traubenik *et al.*, 2020). These studies illustrate the value of INTACT and TRAP in resolving gene regulatory dynamics, ranging from chromatin to translation, at cell-type resolution. Their application to mycorrhizal research, for instance using AM-inducible promoters, could help pinpoint when and where gene regulation occurs during colonisation, and identify specific root cell states involved in symbiosis.

### High-resolution microscopy and spatial complementation to resolve target gene functionality

The high cost of single-cell and spatial omics makes it impractical to generate complete time-resolved maps of gene or protein expression across all tissues, developmental stages, and conditions. In contrast, transgenic transcriptional or translational reporters, though labour-intensive to produce, enable detailed characterization of specific targets under diverse conditions. Classical transcriptional reporters have been successfully applied as high-resolution spatiotemporal biosensors in plant-microbe interactions. For instance, nuclear-localised triple Venus driven by pathogen-associated molecular pattern (PAMP) inducible promoters allowed elucidation of the variable immune responsiveness of differing cell-types and root zones to the PAMP flg22 (Zhou *et al*., 2020; Emonet *et al*., 2021).

Notably, next-generation live-cell imaging technologies allow for a real time-resolved *in vivo* visualisation of reporters. For example, tools such as the AMslide enable the co-cultivation and non-invasive live imaging of diverse plants colonised by AMF, generating timelapses of hyphal growth into the root and arbuscule formation at unparalleled resolution (McGaley *et al.*, 2024). Furthermore, fixation and clearing techniques that reduce autofluorescence, such as ClearSee, preserve fluorophores and can be combined with histological stains, meaning that high-resolution images, penetrating through many cell layers, can now be obtained (Ursache *et al.*, 2018). The combination of whole-mount single-molecule Fluorescent *in-situ* hybridization (FISH) with fluorophore conservation via ClearSee allows the simultaneous visualisation of mRNA at the single-molecule level, as well as protein localisation or promoter activity (Zhao *et al.*, 2023). Furthermore, genetically encoded biosensors can reveal the spatiotemporal patterning of phytohormones and other small molecules (Chen *et al.*, 2024) like auxin (Herud-Sikimić *et al.*, 2021), gibberellin (Rizza *et al.*, 2017), phosphate (Zhang *et al.*, 2022), or calcium (Ca^2+^) (Sun *et al.*, 2015). These have already been extensively applied to plant-microbial interactions, for example the Ca^2+^ FRET (Förster Resonance Energy Transfer) biosensor Cameleon for nuclear calcium spiking in AMS and RNS (Sun *et al.*, 2015; Li *et al.*, 2022), gibberellic acid FRET biosensors GPS1 (Gibberellin Perception Sensor 1) in soybean (Chu *et al.*, 2022) and GPS2 in *Medicago* nodules (Drapek *et al.*, 2024), and phosphate FRET biosensor FLIPPi (FLuorescent Indicator Protein for inorganic Phosphate) in *Brachypodium distachyon* mycorrhizal roots (Zhang *et al.*, 2022).

Transcriptional reporters, translational fusions, and high-resolution microscopy can be used in combination with tissue-specific complementation to resolve the spatial functionality of genes of interest. This approach was applied to the study of pattern-triggered-immunity by Emonet *et al.,* 2021, using a combination of PAMP-triggered transcriptional reporters and tissue-specific promoters driving expression of PAMP receptors to demonstrate the importance of spatially restricted immune responses, as expression of the PAMP receptor in the meristem cortex created hypersensitive plants whose meristems collapsed upon flg22 stimulation (Emonet *et al.*, 2021). The tissue-specific expression of reporter genes and proteins of interest, combined with single-cell omic data, would provide both spatiotemporal context and functional validation, crucial for the characterisation of key pathways underpinning arbuscular mycorrhizal symbiosis.

## Conclusion

The interaction between plant roots and microbial organisms in the rhizosphere results from remarkable precision in the spatio-temporal coordination of genetic programs across a variety of levels, from individual cells to root tissues, and from the epigenetic landscape to post-translational modifications. Furthermore, in natural environments, plant roots face a range of pathogenic and symbiotic microorganisms, requiring not only specific, but also spatially restricted, responses throughout their development and life cycles. Therefore, to better understand how plants interact with their biotic partners, it is essential to take into account the spatial context where these interactions occur. This aspect of plant-microbe interactions is currently at the forefront of research thanks to the advent and expansion of spatially resolved omics.

Mycorrhizal associations present a remarkable model system for study of plant-microbe interactions, being highly ubiquitous, having significant ecological relevance, as well as relying on substantial levels of anatomical and physiological change in both host and invader, thus requiring similarly substantial remodelling at the epigenetic, transcriptomic, and protein levels. Despite much accumulated knowledge in the field, the spatio-temporal nuances of the interaction are still mostly a mystery.

As the arsenal of technologies keeps expanding, the biggest opportunities lie in the ability to combine these diverse techniques using a complementary approach tailored to the specific biological question in hand. Only then is it possible to start piecing together how this intimate interaction is regulated, developed, maintained, and, if necessary, terminated, in all the relevant scales: cell, root, root system and the whole plant.
